# Editorial: Drug Delivery System Based on Nanoparticles for Inflammation and Cancer Therapy

**DOI:** 10.3389/fmolb.2022.938348

**Published:** 2022-05-27

**Authors:** Mingxin Zhang, Ning Lu, Qian Li, Manli Cui, Mingzhen Zhang

**Affiliations:** ^1^ Department of Gastroenterology, The First Affiliated Hospital of Xi’an Medical University, Xi’an, China; ^2^ Shaanxi University of Traditional Chinese Medicine, Xianyang, China; ^3^ School of Basic Medical Sciences, Xi’an Jiaotong University Health Science Center, Xi’an, China

**Keywords:** drug delivery system, nanoparticles, vaccine, inflammation, cancer, therapy

Inflammation is part of the complex biological response of body tissues to harmful stimuli, such as pathogens, damaged cells, or irritants. Cancer is a group of diseases affecting abnormal cell growth to invade or spread to other body parts. When inflammatory responses become chronic, cell mutation and proliferation can result, often creating an environment conducive to cancer development.

Despite many advances in therapeutic strategies against these diseases, their medical applications have been seriously restricted by problems with long-term therapeutic efficacy and unwanted adverse effects. Nanoparticles and vaccines have significant advantages such as high antigen loading efficiency, good targeting, low toxicity, high stability, and diverse administration methods. Therefore, these novel approaches will have significant superiority in inflammation and cancer treatment. Nanoparticle-based drug delivery systems have recently gained attention for inflammation and cancer treatment, and to a certain extent, success has been achieved in the pre-clinical study. However, for clinical translational application, numerous issues have to be addressed in the future, including: 1) the ability to yield a high local drug concentration at the disease site, which should yield prolonged pharmacological activity and maximize drug efficacy; 2) the ability to prevent or reduce the likelihood that a drug will be degraded and lose its efficacy before reaching the site of action; and 3) the potential to reduce dosing frequency and minimize systemic side effects.

The Research Topic entitled “*Drug Delivery System based on Nanoparticles for Inflammation and Cancer Therapy*” presents a series of articles that highlight the advanced studies and strategies that overcome current obstacles in treating inflammation and cancer. The issue is comprised of 6 selected peer-reviewed manuscripts (for original research articles and two reviews) derived from the fields of biomaterials science, nanomedicine, and biology.

Several novel drug delivery systems based on nanoparticles were presented through original research works, including biomimetic nanovesicles (Rampado et al.), implantable bioresponsive hydrogel (Fu et al.), and glutathione-responsive theranostic system (Zheng et al.). These smart delivery systems with tailor-made characteristics have been carefully designed, offering novel conceptual frameworks for anti-inflammatory and antineoplastic applications. In the work of Rampado et al., biomimetic, leukocyte-mimicking nanovesicles (Leukosomes) were developed and presented excellent biocompatibility, endothelium adhesion capability, and tumor target in three dimensional (3D) settings using CRC cell lines. Fu et al. developed an implantable drug loading system, sunitinib nanoparticles @ matrix metalloproteinases -response hydrogel (NSMRH). Their results showed that NSMRH combined with radiotherapy could more effectively control the recurrence of subcutaneous xenograft tumors, prolong the survival time, and have no obvious toxicity in nude mice. Zheng et al. constructed a glutathione- (GSH-) responsive theranostic system (RIF@Cy5.5-HA-NG) for tuberculosis (TB) by photo click reaction-actuated hydrophobic-hydrophobic interaction. This theranostic system achieved the early diagnosis of TB through granulomas-tracking, realized the targeted TB therapy, and provided an especially accurate treatment mapping for TB.

Besides these intelligent drug delivery systems, the final original research presented on this topic by Wang et al. is related to the role of dCTP pyrophosphatase 1 (DCTPP1) in oxidative stress and cisplatin response of ovarian cancer. The results indicated that cisplatin-induced ROS generation could upregulate DCTPP1 expression, showing DCTPP1 involved cisplatin resistance. Therapeutic approaches targeting DCTPP1 may be useful in the treatment of ovarian cancer.

This topic also provides two review papers that summarize “Metal-Organic Frameworks (MOFs) Towards Biomedical Applications” and “Targeting Nutrient Dependency in Cancer Treatment”. Ma et al. outlined the applications of MOFs and their composites in nanomedicine in the last 5 years, and this review emphasized the strategies/function-orientated therapeutics, imaging, sensors, and theranostics of MOFs and their composites. Fan et al. focused on the metabolic reprogramming of tumors. They discussed the existing applications and strategies to target nutrient dependency in different cancer types, accompanied with remaining challenges to further exploit these metabolic vulnerabilities to improve cancer therapies.

To summarize, the role of nanotechnology in inflammation and cancer research has grown dramatically in recent years. We hope that the state-of-the-art articles and reviews collected on this Research Topic highlight the advanced studies and strategies that might overcome current obstacles in treating inflammation and cancer.

